# Automation and Computerization of (Bio)sensing Systems

**DOI:** 10.1021/acssensors.3c01887

**Published:** 2024-02-16

**Authors:** Chamarthi Maheswar Raju, Decibel P. Elpa, Pawel L. Urban

**Affiliations:** Department of Chemistry, National Tsing Hua University 101, Section 2, Kuang-Fu Rd., Hsinchu 300044, Taiwan

**Keywords:** automation, biosensor, computerization, flow injection analysis, microfluidics, prototyping, robotization

## Abstract

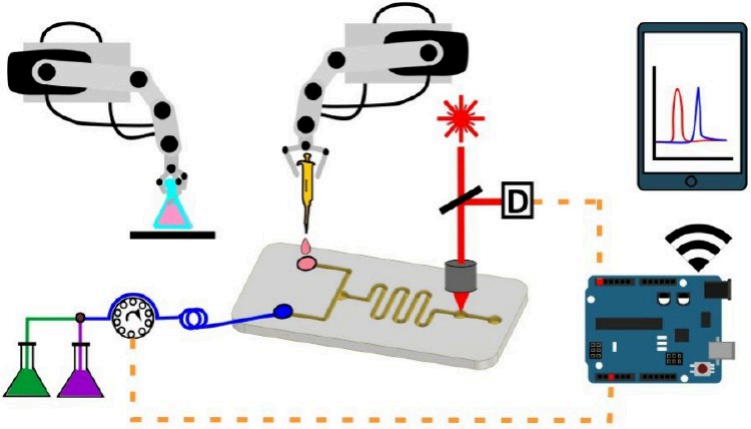

Sensing systems necessitate automation to reduce human effort, increase reproducibility, and enable remote sensing. In this perspective, we highlight different types of sensing systems with elements of automation, which are based on flow injection and sequential injection analysis, microfluidics, robotics, and other prototypes addressing specific real-world problems. Finally, we discuss the role of computer technology in sensing systems. Automated flow injection and sequential injection techniques offer precise and efficient sample handling and dependable outcomes. They enable continuous analysis of numerous samples, boosting throughput, and saving time and resources. They enhance safety by minimizing contact with hazardous chemicals. Microfluidic systems are enhanced by automation to enable precise control of parameters and increase of analysis speed. Robotic sampling and sample preparation platforms excel in precise execution of intricate, repetitive tasks such as sample handling, dilution, and transfer. These platforms enhance efficiency by multitasking, use minimal sample volumes, and they seamlessly integrate with analytical instruments. Other sensor prototypes utilize mechanical devices and computer technology to address real-world issues, offering efficient, accurate, and economical real-time solutions for analyte identification and quantification in remote areas. Computer technology is crucial in modern sensing systems, enabling data acquisition, signal processing, real-time analysis, and data storage. Machine learning and artificial intelligence enhance predictions from the sensor data, supporting the Internet of Things with efficient data management.

According to the International Union of Pure and Applied Chemistry (IUPAC), “automation” is defined as “mechanization with process control, where process means a sequence of manipulations”.^1^ Modern analytical systems are automated to a varied extent and on different levels, including hardware and software components. Thus, the exact meaning of “automation” throughout the literature in the analytical systems field is not consistent. Automated sensing systems can be developed thanks to the progress in various fields, especially electronics, computer science, and robotics.

The earliest computers, such as the Electronic Numerical Integrator and Computer operated in the 1940s, were massive and used vacuum tubes for processing.^2,3^ The invention of transistors in 1947, led to smaller, more reliable, and energy-efficient computers.^4^ In the late 1950s, the development of integrated circuits allowed multiple transistors and electronic components to be condensed onto a single chip, sparking the digital revolution and shifting from mechanical and analog technologies to digital ones.^5^ It was a major milestone in the history of computing, leading to the development of smaller, more powerful, and affordable computers. The integrated circuits have had a significant impact on analytical chemistry by enabling high-level automation, interfacing, integration, miniaturization, portability, real-time data analysis, user-friendly interfaces, cost-effectiveness, customization, connectivity, and data transfer.^6−9^ They revolutionized traditional laboratory analytical methods leading to a widespread adoption of electronic units to minimize human errors and enhance analytical precision.^10^ The integrated circuits also enabled the creation of new types of smart instruments. However, the golden era of automation started in the 1970s with the introduction of microprocessors.^11^ The inventions in electronics were paralleled by the inventions in fluidic systems for handling samples in chemical analysis. Notably, in mid-1950s, Skeggs developed a continuous flow analysis instrument (autoanalyzer) to carry out colorimetric detection of urea and glucose in clinical samples.^12^

Chemical sensors or biosensors are used to detect and quantify specific analytes, including biomolecules.^13^ Automating these sensing devices provides superior control over the experimental conditions—such as temperature, pH, liquid handling, and mixing of reagents and samples—which impact the obtained results. Notably, with the automation of chemical sensors and biosensors, users can rapidly process a large number of samples. This frees them up, so that they can focus on more creative and strategic tasks. This possibility offers increased productivity throughout the analytical workflow. Moreover, automated devices can be used 24/7 without any operator fatigue by making use of suitable algorithms or software to process and analyze the collected sensor data. These algorithms or software programs can provide assistance in carrying out calibration, statistical analysis, and pattern recognition, to extract meaningful information and insights from the collected data.^14^ Through this automatic data processing, one can respond to changes in results in real time, and improve the quality of analytical work.

In the past few years, several reviews have been published discussing the advancements in specific sensing technologies (e.g. refs (15−20)). Our primary focus is to discuss the methodologies for the automation and computerization of various (bio)sensing systems. We aim to offer readers an overview of diverse sensing systems integrated with elements of automation and their associated advantages. Moreover, we would like to expound upon the applications and the manner in which automated setups facilitate the analysis of various types of analytes. We have selected examples of sensing systems, which—based on our judgment—illustrate the automated features to a great extent. In this perspective, sensing systems with elements of automation have been structured into four sections. Initially, we describe the automation of solvent delivery through methodologies such as flow injection and sequential injection analysis. Subsequently, we detail the automation of fluid handling, particularly focusing on microfluidics. Following this, we delve into the automation of sample handling employing simple robotics. The fourth section delineates various prototypes of automated sensing systems. We also examine the role of computer technology within sensing systems.

## Different Types of Sensing Systems with Elements of Automation

### Automation of Solvent Delivery Using Flow Injection and Sequential Injection Analysis

The process of chemical analysis includes sequential stages of liquid handling, analyte detection, data collection, and subsequent calculation of results. To automate the liquid handling process in analytical laboratories, Růžička and Hansen invented the flow injection analysis (FIA) system in 1975.^21^ FIA involves the continuous flow of samples through a system of tubing and various components. It typically uses a peristaltic or syringe pump to propel the sample and reagents through the system, which eliminates the need for manual pipetting of individual samples and reagents. Instead, the sample is introduced into the system through an injection valve, and the system carries out the necessary chemical reactions and measurements. However, the sequential injection analysis (SIA) system was first reported by Růžička and Marshall in 1990 as an extension of FIA.^22^ In SIA, samples and reagents are injected sequentially into a reactor/detector system.^23^ Each injection occurs one after another, controlled by solenoid valves, allowing for sequential chemical reactions, sample dilutions, and other processes within a single analytical run.^24^ By employing the flow-based techniques, metering of samples and reagents is automated. This enables dramatic increase of analytical throughput as compared with manual assays. Moreover, FIA and SIA minimize human error and variabilities associated with manual pipetting, thus enhancing precision in analyses. The high throughput is particularly useful in applications where time is a critical factor, such as clinical diagnostics,^25−27^ environmental sample analysis,^28−31^ food analysis,^32,33^ and pharmaceutical analysis.^34,35^ Nonetheless, the flow-based techniques do not normally address the initial steps of analytical workflows such as sampling and sample preparation.

**Figure 1:**
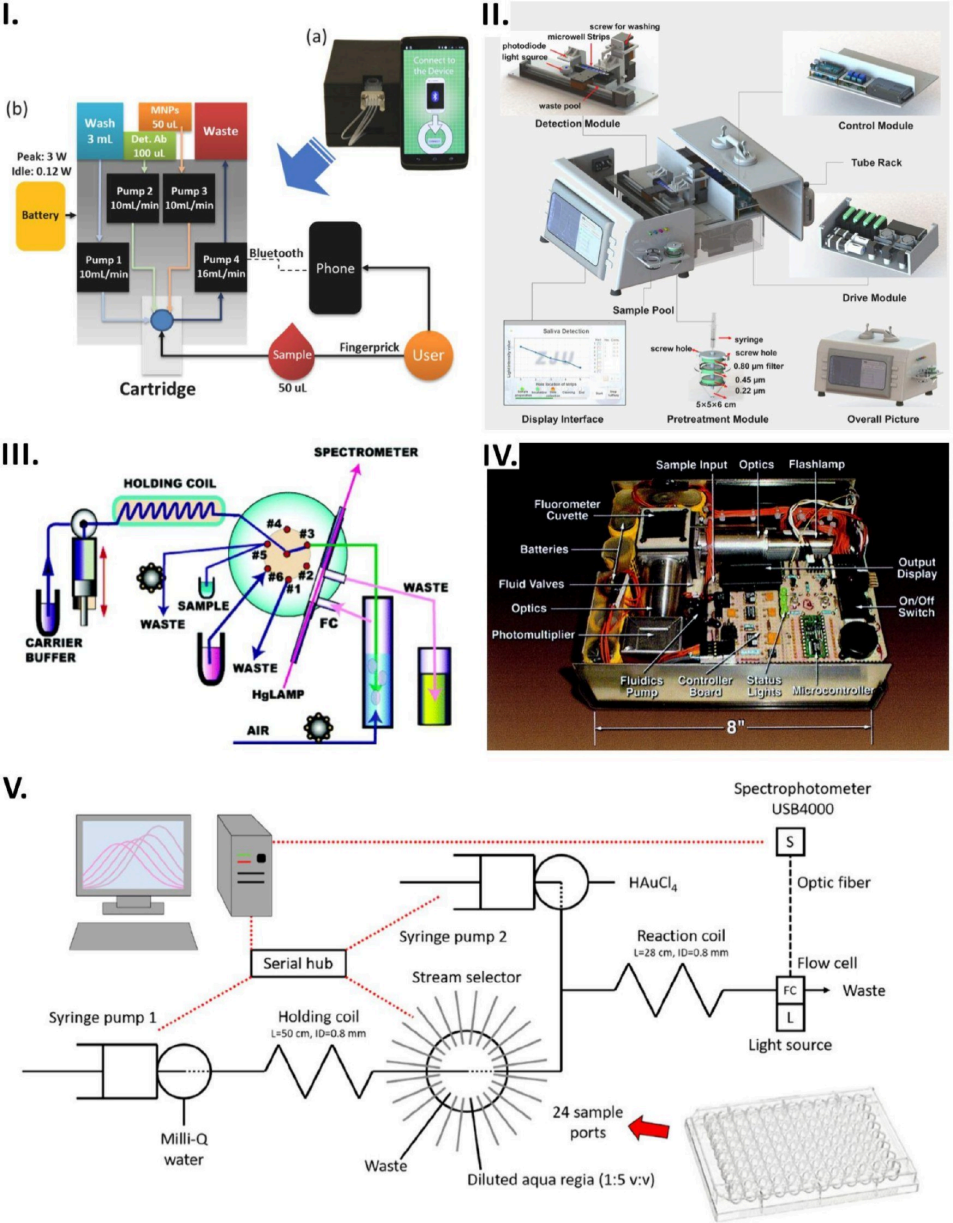
Automated FIA and SIA systems coupled with various sensors and spectrometers. (I-a) A depiction of the automated and mobile magnetoresistive biosensor system. (I-b) Illustration of the system block. Reprinted from *Biosensors and Bioelectronics*, 202, Yao, C.; Ng, E. An Automated and Mobile Magnetoresistive Biosensor System for Early Hepatocellular Diagnosis. 113982, Copyright (2022), with permission from Elsevier.^40^ (II) The comprehensive structural diagram of the designed FASA. Reprinted from *Biosensors and Bioelectronics*, 222, Wang, X.; Sun, X.; Ma, C.; Zhang, Y.; Kong, L.; Hu, Y.; Wan, H.; Wang, P. Multifunctional AuNPs@HRP@FeMOF Immune Scaffold with a Fully Automated Saliva Analyzer for Oral Cancer Screening. 114910, Copyright (2023), with permission from Elsevier.^41^ (III) The schematic diagram of automated sequential injection, lab-on-valve miniaturized system. Reprinted with permission from ref (46). Copyright 2005 American Chemical Society. (IV) Photograph of immunochemical based capture, purification, and detection process. Reprinted from *Biosensors and Bioelectronics*, 14, Carlson, M. A.; Bargeron, C. B.; Benson, R. C.; Fraser, A. B.; Phillips, T. E.; Velky, J. T.; Groopman, J. D.; Strickland, P. T.; Ko, H. W. An Automated, Hand-held Biosensor for Aflatoxin. 841–848, Copyright (2000), with permission from Elsevier.^54^ (V) A graphical representation of automatic flow-through system high throughput ultra sensitive detection of DCF in seawater, utilizing plasmonic nanoparticles probes following ELISA. Reprinted with permission from ref (57). Copyright 2019 American Chemical Society.

The automated FIA/SIA employs distinct categories of sensors or detectors for the examination of various analytes, such as biological, chemical, and elemental. The prominent types of biosensors—utilized in clinical diagnostic automated FIA/SIA—are enzyme-based,^36^ immunological,^37^ and nucleic acid–based.^38^ For instance, immunological biosensors—such as enzyme-linked immunosorbent assay (ELISA)—require meticulous pipetting and manipulation of reagents and samples at every step: coating of sensors with antibodies, blocking, washing, introduction of samples, addition of detection antibodies, incubating and washing, addition of enzyme conjugates, inclusion of substrates, and introduction of stopping solution.^39^ During each individual step, analysts must ensure precise and accurate execution of steps to obtain reliable results. Automation of these steps by FIA/SIA reduces the variabilities caused by manual pipetting and manipulation of reagents and samples. For example, Yao et al. developed an automated mobile magnetoresistive immunoassay biosensor for the early detection of hepatocellular carcinoma in humans.^40^ In this biosensing system, four miniature peristaltic pumps were used to transfer reagents and solutions (Figure 1-I-a,b). These pumps were triggered using a smartphone application (Figure 1-I-b). Then, a signal was transmitted from the smartphone to the microcontroller unit to perform action sequences. In another work, Wang et al. developed a fully automated saliva analyzer (FASA) for the noninvasive detection of Cyfra21–1 biomarker (Figure 1-II).^41^ Cyfra21–1, the soluble fragment of cytokeratin 19, is a potential biomarker for the detection of oral cancer.^42^ In the process of identification of Cyfra21–1 using commercial ELISA, saliva sample pretreatment steps such as centrifugation and filtration need to be performed, which requires specialized equipment and manpower. Using the FASA, sample pretreatment and detection can be performed with minimum human involvement. Intensive insulin therapy is a treatment approach that involves the control of blood glucose levels in critically ill patients, typically those in intensive care units. In this therapy, it is required to determine glucose concentration levels in venous blood for a specific time, typically every 15 to 30 min. The procedure involves labor-intensive tasks such as venipuncture, collection of venous blood, transport of samples to the laboratory, and testing. To minimize these tasks, Schaller et al. developed an automated enzyme-based biosensor for the determination of glucose in venous blood in humans.^43^ Traditional methods for analysis of environmental and food samples often involve manual sample preparation, iterative detection, and other time-consuming procedures. Alternatively, FIA/SIA systems provide high throughput, automate sample preparation, and reduce sample and reagent consumption. For example, the conventional analysis of mercury in environmental and food samples using cold vapor atomic absorption spectrometry (CV-AAS) requires large sample volumes and reagents (2–100 mL), and incurs exposure to hazardous acids, bases, and oxidizers during the sample preparation procedure.^44,45^ To automate this traditional method, Erxleben and Růžička developed a miniaturized and automated sequential injection system for mercury analysis by CV-AAS utilizing microliter volumes of sample and reagents (Figure 1-III).^46^ In this configuration, the stepper motor-driven syringe pumps deliver borohydride reagent solution, hydrochloric acid, and mercury sample into the gas-expansion separator with the aid of a six-position valve (Figure 1-III). However, Komaitis et al. presented a fully automated flow injection analyzer relying on bioluminescent biosensors for the concurrent identification of three heavy metals (Pb^2+^, Hg^2+^, Cu^2+^).^47^ Furthermore, Cocovi-Solberg et al. have devised an automatic three-dimensional (3D)-μFIA platform integrated with a lab-on-valve system.^48^ They have showcased its capability for online mixing with liquid enzymes, employing disposable micro solid-phase-based cleanup for phospholipids, and enabling automatic membrane permeation with potential multiplexed detection.^48^

In contrast to the challenges posed by traditional methods of sample preparation and detection, a range of innovative and automated analytical systems have emerged to streamline these processes, offering greater efficiency and reliability. Thin-layer chromatography, gas chromatography, and high-pressure liquid chromatography are reliable conventional chromatographic methods especially for aflatoxin detection and quantification.^49^ A variety of agricultural crops commonly contain aflatoxins, which are potent carcinogens produced by fungi.^50^ Nevertheless, the purification of aflatoxins from the sample matrix is a prerequisite before utilizing the conventional chromatographic techniques, posing challenges in terms of labor, time, and equipment costs. In recent decades, immunochemical techniques including radioimmunoassay, immunoaffinity column assay, and ELISA have been employed for the purification of aflatoxins.^50−53^ These methods facilitate rapid sample preparation, utilize smaller equipment than the conventional methods, and incur low maintenance cost. However, they are still labor-intensive. To minimize the labor-intensive tasks in aflatoxin analyses, Carlson et al. developed a fully automated immunoaffinity-based hand-held biosensor for aflatoxin detection, which requires 1 mL sample volume and achieves a detection limit of 0.1 parts per billion (ppb) (Figure 1-IV).^54^ By using peristaltic pumps and valves, the automated device guarantees accurate delivery of phosphate-buffered saline, elution fluid, and samples to the affinity columns according to predetermined time sequences. Organic pollutants—such as petroleum hydrocarbons, pesticides, polycyclic aromatic hydrocarbons, polychlorinated biphenyls, pharmaceutical products, and personal care products—can be found in rivers, ponds, and seawater due to various human activities and natural processes. Detecting and quantifying these pollutants often requires sample treatment—such as sample cleanup and preconcentration using solid phase extraction—due to their concentrations in the ppb to parts per trillion range.^55,56^ To circumvent these sample treatment steps, Kaewwonglom et al. developed a plasmonic ELISA biosensor based on an automatic flow-based sensing platform for the detection of diclofenac (DCF) in seawater samples (Figure 1-V).^57^ The aforementioned automated flow-based systems showcased the capability of ELISA biosensors to detect a single analyte across different sample matrices. Furthermore, it is also feasible to concurrently detect multiple analytes. As an example, Knecht et al. devised an automated microarray system that enabled the simultaneous detection of antibiotics in milk through the utilization of an indirect ELISA biosensor.^58^ Moreover, Mishra et al. developed a flow-based biosensor, capable of detecting organophosphate pesticides in milk, through the utilization of a genetically modified acetylcholinesterase enzyme.^59^ In the above-mentioned methods, analytes are detected from liquid samples. To detect analytes in the air, Hindson et al. developed an automated sample preparation method for an environmental monitoring system capable of detecting aerosolized biowarfare agents.^60^ This SIA-based system effectively interfaces aerosol sampling with multiplexed microsphere immunoassay-flow cytometric detection without the need for complex auxiliary hardware.

FIA and SIA offer precision and accuracy in sample handling due to controlled and automated processes, minimize human errors, and ensure reliable results. In addition, these techniques enable rapid and continuous analysis of multiple samples, leading to a substantial increase in the overall throughput. Automated FIA and SIA save time and resources through minimized human intervention and streamlined workflows. Furthermore, utilization of automated FIA and SIA systems reduces sample and reagent consumption, and also provides safety by minimizing direct contact with hazardous chemicals. Limitations of these techniques include limited flexibility in terms of altering predefined sequences, and requirement for proper cleaning, while special precautions are necessary to prevent carryover effects. In addition, automated FIA and SIA systems focus on liquid handling, and cannot easily accommodate nonliquid samples, which can be addressed by robotic sampling systems.

### Automation of Fluid Handling Using Microfluidics

Microfluidics is an interdisciplinary field that focuses on the manipulation and control of small volumes of fluids, typically ranging from 10^–9^ to 10^–18^ L, within microscale devices.^61,62^ Microfluidic devices control flow of fluids using channels, valves, pumps, and other components fabricated on a microscale. Additionally, these devices enable the integration of multiple laboratory functions, such as sample preparation, mixing, separation, and detection, into a compact and portable format. Based on their designs and applications, microfluidic devices fall into distinct categories, including continuous-flow microfluidics,^63^ digital microfluidics,^64^ paper-based microfluidics,^65^ magnetic microfluidics,^66^ open microfluidics,^67^ and droplet-based microfluidics (DBM).^68^ In addition, micro total analysis systems (μTAS) emerged as an alternative option for traditional analytical methods as they enable the processing of fluids in microchannel structures at the microliter level to perform complete chemical analyses.^69,70^ The automation potential and portability of μTAS are unique properties inherent in these systems, making them a viable choice for analyzing complex samples efficiently with minimum cost, energy, and chemical consumption.^70^

**Figure 2:**
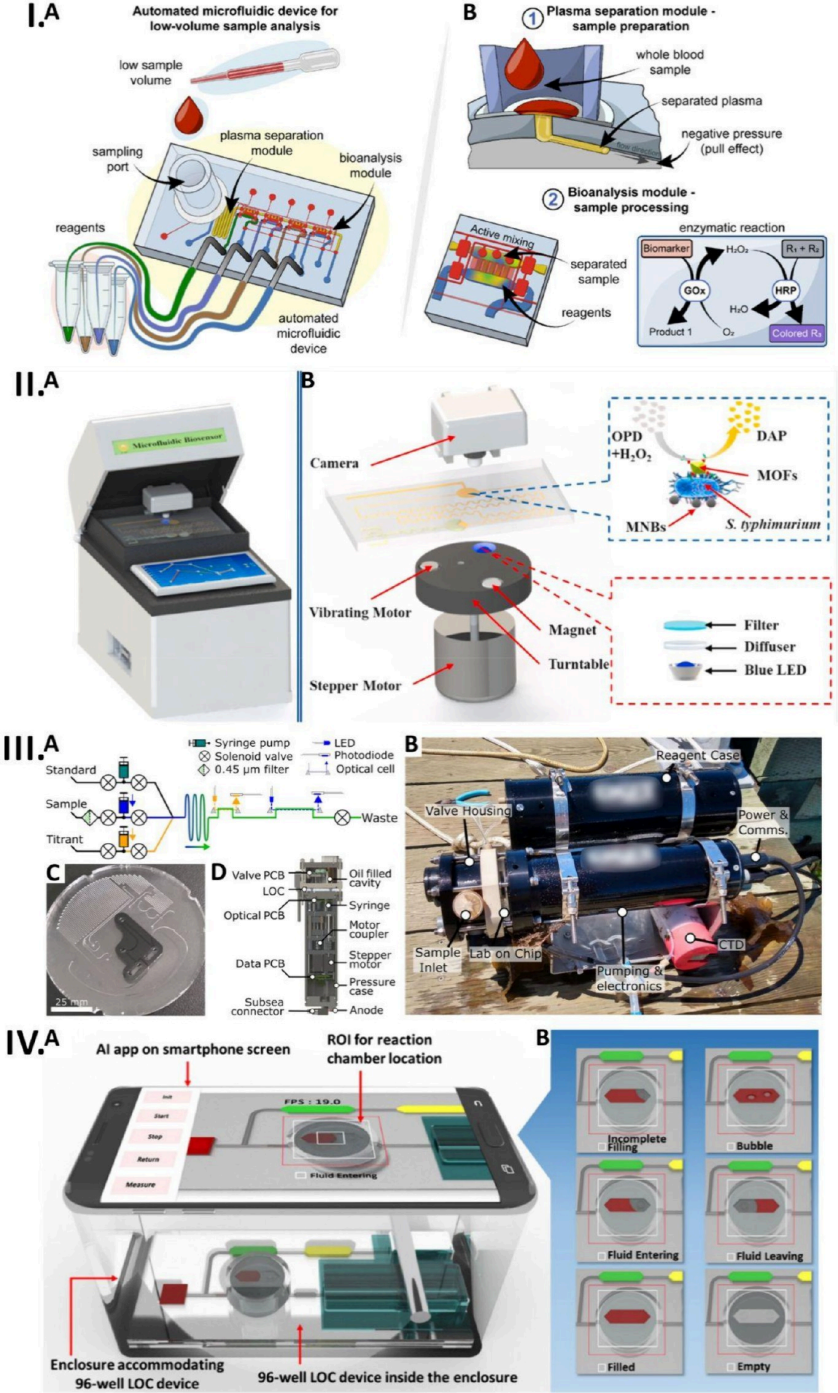
Automated microfluidics devices. (I-A) A microfluidic device consisting of plasma separation and bioanalysis module has been developed to facilitate on-chip collection of plasma and glucose detection. (I-B) Schematic representation of plasma collection module, integrated microvalves within each analysis unit, and colorimetric detection of glucose. Reprinted with permission from ref (73). Copyright 2022 American Chemical Society. (II-A) The microfluidic biosensor prototype for the detection of *Salmonella* pathogenic bacteria. (II-B) The concept and design of microfluidic biosensor for the detection of *Salmonella* pathogenic bacteria. Reprinted from *Biosensors and Bioelectronics*, 178, Qi, W.; Zheng, L.; Wang, S.; Huang, F.; Liu, T.; Jiang, H.; Lin, J. A Microfluidic Biosensor For Rapid and Automatic Detection of *Salmonella* Using Metal–Organic Framework and Raspberry Pi. 113020, Copyright (2021), with permission from Elsevier.^80^ (III-A) A schematic representation of pumps, valves, optical cells, and optical components. (III-B) A photograph of an alkalinity analyzer. (III-C) Image of lab-on-chip platform. Reprinted with permission from ref (85). Copyright 2023 American Chemical Society. (IV-A) AI application is displayed on the smartphone screen to control the microfluidic chip. (IV-B) Images of different filling states of the reaction chamber were captured using a smartphone. Reprinted with permission from ref (85). Copyright 2023 American Chemical Society.

Microfluidic systems provide improved accuracy and efficiency of analyses. These microfabricated devices have been suitable for applications wherein rapid measurements of minute samples are required, such as in the areas of medical diagnostics, food analysis, and environmental analysis. The commercially available electrochemical glucometers are capable of analyzing the glucose levels in whole blood samples and provide a numerical value within seconds. However, they exhibit inaccuracies when analyzing glucose levels <2 mM due to the inherent variability in hematocrit levels.^71−73^ Traditionally, centrifugation has been employed for separating plasma from whole blood samples. Nevertheless, in recent years, microfluidic devices emerged as a viable technique for separating plasma from whole blood, particularly in small volumes.^74,75^ An integrated on-chip plasma separation module (PSM) for an automated microfluidic device was developed by Gonzalez-Suarez et al.^73^ The PSM enables the analysis of glucose levels in microliter volumes of blood (Figure 2-I-A,B). Plasma flows into the microchannel, following its separation in the PSM. The analytical workflow for plasma separation and biomarker analysis was controlled by six onboard microvalves. Each microvalve was connected to a computer-controlled external three-way solenoid valve to regulate the pressure (Figure 2-I-B). When the solenoid valves are activated, microvalves are pressurized to impede the flow within the microchannels. To facilitate active mixing of plasma and reagents, the microvalves are sequentially activated and deactivated to induce mixing (Figure 2-I-B). Determination of glucose concentration was accomplished through the execution of on-chip enzymatic colorimetric assays.

Detection of *Salmonella* in food is essential for guaranteeing food quality and ensuring compliance with the regulations.^76^ Traditional culture methods for *Salmonella* detection in food samples typically require 4–5 days, while also entailing a large amount of labor.^77,78^ The contemporary methodologies such as ELISA, and polymerase chain reaction (PCR) involve complex sample pretreatment procedures and incur long analysis times, ranging from 12 to 24 h.^77,79^ However, with the advent of microfluidic technology, the analysis time for *Salmonella* detection can be reduced. Qi et al. developed an automatic microfluidic biosensor for the detection of *Salmonella* employing metal–organic frameworks and Raspberry Pi, which reduced the analysis time to 1 h (Figure 2-II-A,B).^80^ In this setup, the accurate control of solution pumping, mixing, incubation, washing, reactions, and separation of bacteria is achieved through a custom application running on the Raspberry Pi single-board computer (Figure 2-II-A,B). This custom application can also analyze images utilizing the Python OpenCV library.

The analysis of seawater alkalinity is conducted to understand the intricate carbon dioxide cycle dynamics of the ocean, its pivotal role in the global climate system, and the environmental status of marine ecosystems.^81^ Typically, seawater alkalinity has been determined using different types of methods namely potentiometric titration,^82^ spectroscopic,^83^ and coulometric methods.^84^ All these methods require sampling from the ocean and subsequent sample analysis in the analytical laboratories. This process leads to increased duration of analysis and requires a substantial amount of labor. To address this bottleneck, researchers focus on automating real-time seawater alkalinity monitoring, eliminating the need for human involvement. For instance, Sonnichsen et al. developed an automated microfluidic analyzer for in situ monitoring of total alkalinity in seawater (Figure 2-III-A–C).^85^ This device employs custom-developed software to control syringe pumps to inject standard, titrant, indicator, and sample into a serpentine mixer lab-on-chip microfluidic analyzer (Figure 2-III-A). The resulting mixture is then directed into the cells for optical detection at two specific wavelengths. The analysis of the absorbance ratio at these wavelengths enables calculation of the pH value.

Although microfluidic chips are employed diversely to optimize process automation, aiming to reduce human intervention, certain microfluidic chips continue to exhibit deficiencies in terms of reliability and repeatability of outcomes, primarily attributed to factors such as imprecision of flow generation by pump,^86^ bubble formation, as well as inadequate filling of microchannels and the reaction chamber (RC) within the microfluidic chip. A smartphone-operated, artificial intelligence (AI)-controlled microfluidic chip device developed by Bhuiyan et al. addressed microfluidic errors by liquid automation and bubble elimination (Figure 2-IV-A,B).^87^ In this configuration, an AI image recognition application has been implemented in the automated immunosensing platform to identify the incomplete filling of the RC and the generation of bubbles within the RC (Figure 2-IV-B). Besides, AI has been implemented in low-cost hardware, specifically smartphones, employing two functionalities (Haar cascade classifier and AdaBoost machine learning algorithm) to enhance the operation of AI image recognition. Moreover, AI facilitated the quantification of cardiac troponin I biomarker through this automated microfluidic ELISA platform. The high-throughput nature and precise controllability of microfluidic devices enables large and complex data set generation; thereby requiring high level of data processing.^88^ Consequently, microfluidic devices can be integrated with machine learning to obtain valuable and accurate information, paving the way for the development of intelligent microfluidics.^88^ A comprehensive examination of the complex droplet data in DBM is crucial for the identification, categorization, and quantification of the species contained within the droplets. Complex statistical and data analysis including fluid control, droplet size prediction, recognition of flow pattern and identification, as well as droplet classification and sorting within a microfluidic device is automated by implementation of machine learning models.^88,89^

Fabrication of certain microfluidic devices requires careful selection of compatible materials, bonding, and sealing, along with techniques like photolithography or soft lithography.^90,91^ These fabrication procedures involve time-consuming steps and also require clean room facilities. Nevertheless, it is worth noting that some microfluidic devices can also be mass-produced cost-effectively using injection molding techniques, thus reducing the overall cost of these devices.^91,92^ In recent years, there have been developments in nonmicrofluidic methods for manipulating microliters of liquid in an open environment. For instance, Yang et al. developed an acoustic wave-assisted microscale assay platform for manipulating the microliter range volume of the sample and reagent.^93^ In this experimental configuration, a sound intensity gradient is employed to propel the sample and reagent droplets positioned on a hydrophobic thread. Subsequently, migrating droplets merge at the actuation range and progress further for fluorometric detection. In other work, Kiani et al. developed an automated and cost-effective digital microfluidic platform for precise mechanical manipulation of the micro/nanoliter droplets.^94^ This experimental platform incorporates a robotic arm that is equipped with multiple actuators for dispensing and manipulating droplets on a superhydrophobic cartridge. Their system is also integrated with magnetic and heating modules that facilitate particle manipulation and droplet heating. The inclusion of a comprehensive fluidic toolbox and multiple detection options render this platform highly promising as a droplet-based total analysis technology.

Automation has a significant impact on microfluidics-based sensors, revolutionizing the manner in which measurements are conducted and significantly enhancing the capabilities of this technology. Automation enables precise control of fluid flow, temperature, and other experimental parameters, which is crucial in microfluidics, where even minor changes can significantly influence the results. Moreover, automation provides high-throughput analysis, which are particularly valuable in drug discovery, where extensive screening of compounds is necessary. Furthermore, the implementation of computer technologies such as machine learning, computer vision, and AI enhances microfluidic workflows by enabling intelligent microfluidics for improved feedback and controllability of microfluidic platforms.

### Automation of Sample Handling with Simple Robotics

Robotic sampling and sample preparation involve the utilization of robotic systems to automate the acquisition of samples from diverse sources and the subsequent preparation of those samples for analysis.^95,96^ These processes involve the deployment of robots or robotic arms equipped with specialized tools and instruments to execute tasks associated with sample collection, handling, and processing.^97^ Notably, robotic sample preparation provides various advantages over manual methods, including higher efficiency and precision; and reduced human errors. In chemical analysis, robotic systems offer capabilities such as dispensing reagents, mixing, performing serial dilutions, centrifugation, pipetting, labeling, and transferring aliquots between different pieces of labware. These capabilities enable the implementation of robotic sampling and sample preparation in various fields, including bioanalysis,^98^ clinical analysis,^99,100^ pharmaceutical analysis,^101^ food analysis,^102,103^ environmental analysis,^104,105^ and forensic toxicology.^106^

**Figure 3:**
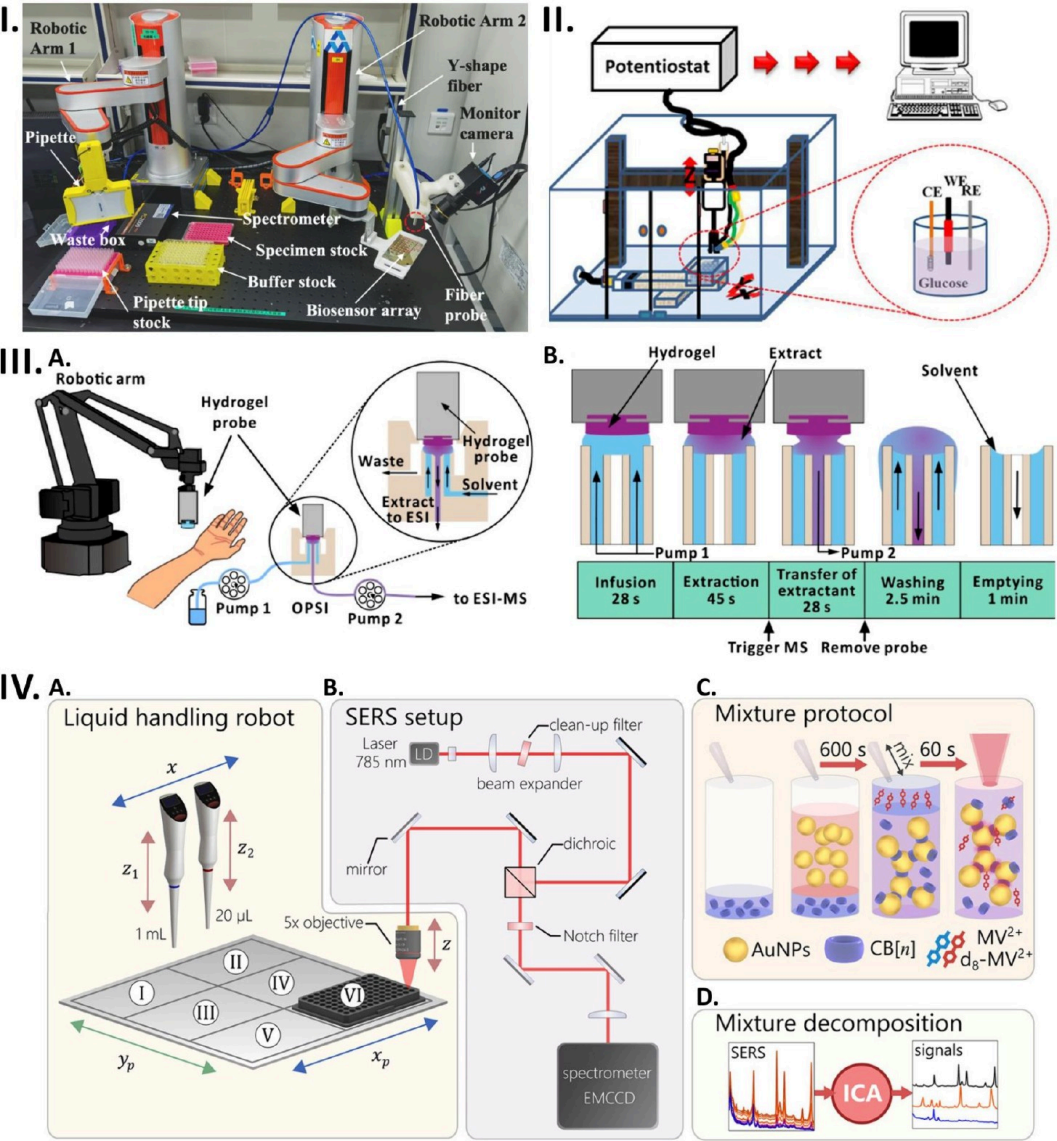
Robotic sampling and sample preparation platforms. (I) Photograph of automatic dual robotic arm high-throughput biosensing platform for COVID-19 detection. Reprinted from *Biosensors and Bioelectronics*, 220, Rong, G.; Zheng, Y.; Li, X.; Guo, M.; Su, Y.; Bian, S.; Dang, B.; Chen, Y.; Zhang, Y.; Shen, L.; Jin, H.; Yan, R.; Wen, L.; Zhu, P.; Sawan, M. A High-Throughput Fully Automatic Biosensing Platform for Efficient COVID-19 Detection. 114861, Copyright (2023) with permission from Elsevier.^110^ (II) Photograph of robotic amperometric enzyme 24-well microplate biosensing platform. Reprinted with permission from ref (112). Copyright 2017 American Chemical Society. (III-A) Schematic diagram of robotic sampling of skin excretion and OPSI extraction setup. (III-B) Illustration of OPSI working principle. Reprinted with permission from ref (116). Copyright 2023 American Chemical Society. (IV-A) Schematic diagram illustration of liquid handling robot with two micropipettes operating on the platform. The platform is divided into six regions each thoughtfully designed to accommodate the multiwell plate, pipet tips, and vials with stock solutions. (IV-B) Illustration of SERS setup. (IV-C) The schematic diagram illustrates the protocol followed by the robot to combine AuNPs and cucurbiturils for substrate preparation, along with the subsequent addition of an analyte. (IV-D) Proposal for the independent component analysis and refining of data into component signals. Reprinted with permission from ref (121). The article is licensed under the CC-BY-NC-ND 4.0 (https://creativecommons.org/licenses/by-nc-nd/4.0/).

With the advancements in robotic technologies, robotic systems are now readily integrated with other equipment and instruments to achieve fully automated sensing systems, from either sample handling or preparation to detection. We have selected examples of these sensing systems, which involve robotic operations to achieve full automation of the analytical sensing workflow. Diverse methodologies exist for detecting the presence of the SARS-CoV-2 virus in human samples. These methodologies encompass PCR, reverse transcription-polymerase chain reaction (RT-PCR), antigen tests, nucleic acid amplification tests (NAAT), clustered regularly interspaced short palindromic repeats (CRISPR)-based tests, computed tomography (CT) scans, and breath tests. Among these approaches, the antigen method stands out for its rapidity and cost-effectiveness, although its sensitivity and specificity are comparatively lower than RT-PCR.^107^ CT scans are often utilized in conjunction with other diagnostic outcomes to assist in the accurate diagnosis of COVID-19.^108^ The methods such as NAAT, PCR, RT-PCR, and CRISPR-based tests are associated with complex sample preparation procedures, possess limited throughput, require clean environment as well as trained personnel, and exhibit limited cost-effectiveness.^109,110^ Rong et al. developed an automated antigen-based biosensing platform with localized surface plasmon resonance detection for COVID-19 to offer enhanced throughput, increased sensitivity, and rapid testing capabilities for SARS-CoV-2 (Figure 3-I).^110^ In this configuration, two robotic arms were utilized for sample loading, incubation, sensor surface rising, and optical measurement using a portable spectrometer. The detection process takes 5 min, rendering it highly appropriate for extensive deployment in rapid and onsite COVID-19 screening.

ELISA is widely used in disease diagnosis, drug testing, environmental monitoring, veterinary diagnosis, and research applications. As previously discussed, traditional ELISA methodologies have often been characterized as laborious, time-intensive, and dependent on the proficiency of technicians. To overcome these limitations, Zhou et al. developed a fully automated robotic ELISA platform to enable a human-free workflow from sample handling to final assay.^111^ This robotic ELISA platform is equipped with a selective compliance assembly robot arm, and a pressure control unit, featuring high-precision solenoid valves. The Python-programmed robotic arm platform facilitates the grab, move, and release of the hybrid microfluidic chip. A set of two 3D-printed robotic microfluidic interfaces (RMI) are mounted into the robotic arm, followed by a connection of the RMI to the pneumatic drive, allowing for precise pressure control. As a result, applying positive and negative pressure to the microfluidic chip from the pneumatic drive is possible. An OpenCV-programmed camera is employed in the robotic arm for real-time colorimetric reading of the detection chamber. The image data is transferred wirelessly and analyzed instantaneously.

Microplate-based assays integrated with automated workstations are effective for streamlining high-throughput data collection and analysis. To create a cost-effective and user-friendly microplate-based biosensor assay, Teanphonkrang et al. devised an automated workstation for the robotic amperometric enzyme biosensing platform of a 24-well microplate electroanalysis (Figure 3-II).^112^ In this robotic workstation, the biosensor, counter, and reference electrode assemblies move over the 24-well plastic microtiter plate in up–down (*Z*), left–right (*X*), and backward–forward (*Y*) directions with the aid of computer-controlled micropositioners with stepper motors. This three-electrode assembly moves from well to well, and dips into each well on arrival to record voltammograms or amperograms at certain time intervals. This microplate biosensor setup improves the throughput and reduces human intervention in analysis. Alternatively, Yang et al. have developed an automated high-throughput microplate reader for rapid colorimetric biosensing.^113^ It comprises a microfiber optic spectrometer, an optical light source, an *x-y* axis two-dimensional (2D) slide table, and a computer with LabVIEW software. The 2D slide table is set in motion by pulses generated from the master control circuit, thereby enabling precise positioning and swift movement of the microplate during colorimetric measurements.

Detection of metabolites and protein breakdown products in human sweat is potentially important for clinical diagnostics. In recent times, biocompatible agarose hydrogel is being utilized for manual sampling of human sweat and skin excretions.^114,115^ However, it should be noted that although manual sample collection from human skin using hydrogels is simple, the desorption or re-extraction of analytes from the hydrogels and its subsequent detection require skill and expertise. In order to address this limitation, Yu et al. developed a vending-machine-style skin excretion sensing platform, which serves to automate the sampling and analysis of human sweat and skin excretion.^116^ In this automatic sensing platform, the robotic arm picks up a hydrogel probe and collects the sample from the forearm (Figure 3-III-A). After that, the robotic arm docks the hydrogel probe in the open port sampling interface (OPSI) (Figure 3-III-B). The sample extracted from the hydrogel probe in the OPSI is transferred to mass spectrometry (MS) interface. In other report, Chiu et al. demonstrated a robotics-assisted mass spectrometry assay (RAMSAY) platform designed to expedite sample delivery, biochemical reaction, and MS analysis.^117^ In the RAMSAY platform, the robotic arm picks up a sample vial after conducting a barcode scan originating from the designated sample drop-off zone. Afterward, the robotic arm initiates the sample processing procedure following a programmed sequence. The sample is immediately conveyed to the Venturi pump inlet, and thereafter, the Venturi pump sprays it in front of the MS orifice. In a follow-up work, Chen et al. developed the RAMSAY-2 platform, featuring dual robotic arms for evaluation of enzymatic activities in samples.^118^ In this platform, robotic arm 1 retrieves the sample from the drop-off zone and transports it to the outlet of the reagent tubing. Subsequently, robotic arm 1 places the vial inside the water bath, which is set to a temperature of 37 °C for incubation. After the incubation step, the sample vial is transferred to the transit platform. Right after that, robotic arm 2 grabs the sample vial from the transit platform and aligns it with the sampling capillary at the ion source inlet for spraying. In general, the analysis of volatile organic compounds (VOCs) requires homogenization and solvent extraction of the sample. Nevertheless, this process does not provide spatial resolution and is also labor-intensive. To analyze the VOCs emanating from solid specimens, Abu Bakar et al. developed a robotic arm-based open-space sampling method.^119^ In this methodology, a vacuum-assisted suction sampling probe is affixed to the robotic arm, which subsequently maneuvers the probe to conduct scans of the flat surface samples. The VOCs—collected by the probe—are then transferred to the atmospheric chemical ionization source of tandem MS to produce chemical maps.

Surface-enhanced Raman spectroscopy is considered a powerful analytical technique for molecular sensing. Raman signals experience substantial enhancement through the nanoassemblies of molecules with metal nanoparticles.^120^ To automate the sample preparation of these nanoassembles, Grys et al. integrated a liquid-handling robot with surface-enhanced Raman spectroscopy (Figure 3-IV-C).^121^ In the presented arrangement, a liquid handling robot is equipped with two single-channel micropipettes to achieve the sample preparation of nanoassembles (Figure 3-IV-A). Further, the platform executes automatic movements in *X* and *Y* directions to precisely position the containers beneath the microscope for surface-enhanced Raman spectroscopy measurements (Figure 3-IV-B). Moreover, independent competent analysis algorithm has been developed to deconvolute the component signals from the data (Figure 3-IV-D).

Robotic platforms can perform complex and routine tasks such as mixing, diluting, and transferring samples in a controlled and precise manner. Robotic platforms can handle multiple tasks simultaneously to increase the throughput and are capable of handling smaller sample volumes efficiently to reduce sample consumption. Moreover, robotic systems can be integrated with various analytical instruments such as mass spectrometry and spectrophotometers to enable highly selective analysis. However, one should consider the initial investment and technical complexity when integrating robotics into various analytical platforms.

### Other Prototypes of Automated Sensing Systems

**Figure 4:**
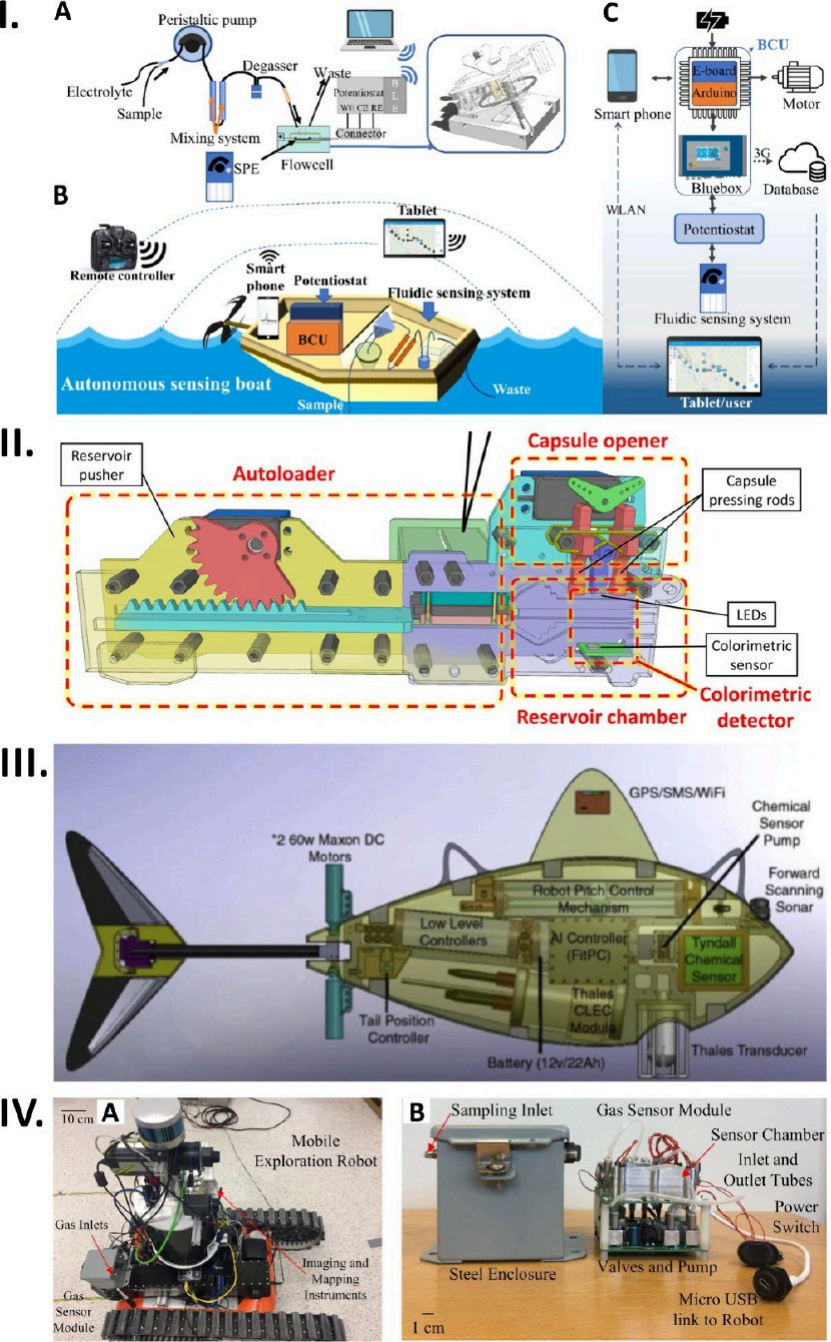
Sensor prototypes for addressing real-world problems. (I-A) Schematic representation of fluid heavy metal sensing system. (I-B) Graphical representation of an autonomous sensing boat with fluid heavy metal sensing system. (I-C) Schematic diagram of an electronic controlling unit of an autonomous sensing boat. Reprinted with permission from ref (130). The article is licensed under the CC-BY 4.0 (https://creativecommons.org/licenses/by/4.0/). (II) Schematic view of an automatic colorimetric sensor for detection of phosphate and nitrite in agricultural water. Reprinted with permission from ref (131). Copyright 2018 American Chemical Society. (III) Schematic diagram of autonomous robotic fish for monitoring port water quality. Reprinted from *Reference Module in Materials Science and Materials Engineering, Comprehensive Materials Processing, 13*, 1^st^ ed., Ogurtsov, V. I; Twomey, K.; Herzog, G. Development of an Integrated Electrochemical Sensing System to Monitor Port Water Quality Using Autonomous Robotic Fish. 317–351, Copyright (2014), with permission from Elsevier.^134^ (IV-A) Photograph of mobile tracking robot for flammable gas sensing. (IV-B) The photograph showcases the steel enclosure and sensor module, showcasing the internal circuitry featuring the gas sensor PCB and control PCB. Reprinted from *Sensors and Actuators B: Chemical*, 279, Vincent, T. A.; Xing, Y.; Cole, M.; Gardner, J. W. Investigation of the Response of High-Bandwidth MOX Sensors to Gas Plumes for Application on a Mobile Robot in Hazardous Environments. 351–360, Copyright (2019) with permission from Elsevier.^137^

Sensor prototypes can help to address analytical challenges in various fields, which include agriculture and soil analysis,^122^ clinical diagnostics,^123^ environmental gas monitoring,^124^ healthcare monitoring,^125^ environmental monitoring,^126^ water quality monitoring,^127^ food safety and quality control,^128^ and air pollution monitoring.^129^ For instance, Yang et al. developed an autonomous sensing boat for on-site heavy metal detection in natural waters utilizing square-wave anodic stripping voltammetry, which can circumvent the need for off-site costly laboratory detection and the time-consuming operations conducted by trained personnel (Figure 4-I-A–C).^130^ In this platform, a natural water sample and electrolyte are first delivered to the mixing system using a peristaltic pump (Figure 4-I-A–C). Afterward, the sample and electrolyte mixture undergoes degassing before entering the flow cell. Following the deposition, equilibration, and stripping in the flow cell, a voltammogram is being recorded using a potentiostat (Figure 4-I-A). Eventually, the recorded voltammogram data are transferred via Bluetooth to the data analyzer (Figure 4-I-C).

From autonomous sensing boats for heavy metal detection to in situ automatic sensors for continuous monitoring of phosphate and nitrite levels in agricultural waters, we can see how sensor prototypes are developed to cater wide range of applications. In a representative work, Lin et al. developed an in situ automatic sensor for the continuous monitoring of phosphate and nitrite levels in agricultural waters (Figure 4-II).^131^ This device incorporates reservoirs with a fish-bite-inspired design for sample collection, followed by chromogenic reactions performed within these reservoirs. Initially, an open fish-bite reservoir is filled with the surrounding water, after which the reservoir autonomously seals itself and is then propelled toward the position of the sensors through the utilization of servo motors. Subsequently, reagents are introduced to the water sample, and colorimetric detection is performed.

Autonomous robots possess the potential to revolutionize multiple facets of the (bio)sensing field, including analytical chemistry. These robots—often equipped with advanced sensors, AI, and machine learning capabilities—adeptly perform analytical tasks with minimal human intervention. Robotic fishes have been developed by various researchers over the years for different applications such as environmental monitoring, scientific research, and underwater exploration.^132,133^ For example, Ogurtsov et al. devised an autonomous robotic fish equipped with electrochemical sensors to monitor port water quality (Figure 4-III).^134^ This innovative robotic fish demonstrated the capabilities to independently navigate in the port waters, and facilitate the collection of data related to the heavy metals, phenols, dissolved oxygen, conductivity, and oxidation–reduction potential. In addition, control and signal processing algorithms have been implemented in the robotic fish.

While autonomous robotic fishes have been designed for underwater applications such as environmental monitoring, automated sensing systems are also deployed on unmanned vehicles. In one representative work, He et al. developed an autonomous aerial robot for chemical sensing in urban and suburban environments.^135^ This aerial robot is equipped with a molecular property spectrometer chemical sensor, enabling it to detect and quantify different flammable gases. Moreover, the robot can scan autonomously and map areas affected by chemical leaks. Notably, it has attributes such as real-time data visualization and collision-free navigation. Moreover, Kostyukevich et al. developed a multicopter mounted with a field asymmetric ion mobility spectrometer and an array of semiconductor gas sensors to detect chemical warfare agents, explosives, and air impurities in hard-to-reach places.^136^ This system enables a 15 min flight and remote access to the acquired data. Furthermore, Vincent et al. have created a mobile exploration robot fitted with high-bandwidth metal-oxide sensors to remotely detect flammable gas plumes in hazardous conditions (Figure 4-IV-A,B).^137^ The mobile robot’s capabilities have been evaluated both in controlled lab conditions and real-world scenarios, enabling it to detect and map the presence of combustible gases. Firefighters can employ this setup to identify hazardous environments during disasters.

Various sensor prototypes effectively tackle real-world problems by providing efficient, accurate, and economically viable real-time solutions for the identification and quantification of substances, even in geographically challenging or remote areas. These prototypes leverage advancements in computer technology, system control, sensor design, and robotics to address the challenges and enhance analytical procedures.

## Computer Technology in Sensing Systems

Computer technology often plays a key role in modern sensing systems. A wide variety of sensors interface with computer devices such as microcontrollers, single-board computers, and conventional computers to convert analog signals into digital raw data to process them further.^10,138,139^ Raw data often contains unwanted noise and irrelevant information. Extraction of significant information from acquired raw data requires the implementation of data processing procedures such as signal amplification, filtering, and noise reduction.^10,140^ Sensors can be combined with processors, and operated with appropriate algorithms, machine learning, and AI to manipulate and analyze the data in real-time, which is crucial for taking timely decisions based on the acquired data.^141−143^ Furthermore, some sensor devices are integrated with the Internet of Things to allow remote monitoring, control, and data analysis.^144−146^

To examine the distribution of substances on the surface, Huang et al. utilized an enzyme-loaded hydrogel array along with a custom-built array reader.^147^ This device employs a Raspberry Pi single-board computer with a miniature camera that captures the color changes in the hydrogel array. Computer vision algorithms are employed to determine the color pixel saturation values of individual hydrogel micropatches. Subsequently, the collected color pixel values are plotted on a heat map to show the distribution of substances on the surface. In other work, a miniaturized hand-held cloud-integrated BioChemPen was devised to detect the chemical residues present on solid surfaces.^148^ In this device, analog signals—generated by the sensor—undergo amplification and subsequent averaging of the signal values to reduce noise. The resultant averaged signal values are subjected to subtraction with baseline values and then converted the negative values into positive values. The acquired data are stored within the text file. This data set is subsequently preserved within the flash memory of the microcontroller board featuring Wi-Fi connectivity, thus enabling the transfer of stored data to the cloud infrastructure for remote data monitoring. In other representative work, Hsu et al. developed a 3D-printed fluorometric probe for monitoring fluorescence chemical reactions.^146^ A Wi-Fi-enabled electronic controller has been incorporated to facilitate the real-time transmission of the sensor data to a cloud-based storage platform. This configuration enables extended and remote monitoring of fluorescent chemical reactions conducted within the laboratory setting.

Machine learning and deep learning are subsets of AI that differ in their approach and complexity.^149^ Machine learning involves the use of algorithms that enable computers to learn from data, identify patterns, and make decisions without being explicitly programmed.^150^ It encompasses a broad spectrum of techniques, including regression, clustering, and decision trees. Deep learning, a specialized field within machine learning, relies on neural networks with multiple layers to learn representation of data, such as images, audio, and text, by automatically discovering intricate patterns through hierarchical layers of neural nodes.^151,152^ While machine learning covers a wide range of algorithms and methodologies, deep learning is a specific type of machine learning that originated from the research of artificial neural network, often requiring a massive amount of data for training and more computational resources.^153^ Moreover, machine learning and deep learning advance the sensing systems by acquiring knowledge from data and generating predictions based on sensor inputs. In recent years, machine learning has found numerous applications in the field of biosensors such as signal processing and noise reduction, pattern recognition, classification, signal drift correction, real-time data monitoring and prediction, feature selection and dimensionality reduction, and anomaly detection.^143,154,155^ For instance, Pennacchio et al. developed a mercury detection biosensor based on hydrophobin chimera, which was enhanced with machine learning.^156^ In this mercury detection platform, machine-learning algorithms such as random forest, multilayer perceptron, and XGBoost are both utilized as classification and regression algorithms to classify and predict the fluorescence intensity and concentration of heavy metals. Furthermore, Guo et al. developed carbon nanotube thin film biosensors for the identification of heart failure.^157^ This study utilized a classification-based machine learning algorithm to aid in the identification process. The application of deep learning techniques in biosensors is gradually expanding. Deep learning is applied to biological data to improve the accuracy and speed of analyzing biological information. It can enhance diagnostic capabilities and real-time monitoring. For instance, liquid crystal sensors are extensively used for the detection of specific gas, chemicals, and biological substances.^158,159^ However, evaluating optical images obtained from liquid crystal sensors is a complex task that demands substantial amount of effort.^160^ To address this concern, Zhang et al. implemented deep learning techniques for the computation of images related to liquid crystal sensing.^160^ In this configuration, liquid crystal images are prepared for training a convolutional neural network. The objective of this training is to enable accurate prediction of positive and negative areas within the liquid crystals, thereby achieving an increased level of precision in computational analyses of optical micrographs obtained from liquid crystal-based sensors. Additionally, Wu et al. developed an automated readout method for aggregation-based assays employing a wide-field lens-free on-chip microscope.^161^ This method possesses the capability to swiftly analyze and quantify 3D microscope particle aggregation events through the utilization of deep learning-based holographic image reconstruction.^161^ Similarly, in other work, Wu et al. formulated an approach to detect label-free bioaerosol by utilizing holographic microscopy and deep learning.^162^

Computer technology serves as an essential component within modern sensing systems, fulfilling indispensable functions such as facilitating data acquisition and conversion, enabling signal processing, and facilitating real-time data analysis. Furthermore, computer technology provides the infrastructure required for efficient data storage, management, and retrieval of data, which conforms to the principles of the Internet of Things. Besides, computer technologies facilitate the development of graphical user interfaces and dashboards for configuring settings and visualizing data.

## Conclusions and Future Perspective

Automation has entered the (bio)sensing field. The common setups include flow injection and sequential injection analysis, microfluidics, robotics, and other prototypes addressing specific real-world problems. Computer technology also plays a role in the automation of sensing systems. Automated sensing systems offer several advantages over manual sensing methods such as increased accuracy and precision, real-time analysis, cost-effectiveness, data integrity, and traceability. However, automated sensing systems are complex devices that require a combination of skills from various fields, including engineering, programming, and data analysis.^163^ Such systems incorporate sensors to collect data from different environments. Therefore, to develop them, a strong grasp of sensing technology is required. Understanding sensor signal processing and elements of electronics is also crucial. These systems generate large data; thus, there is a need for programming skills to perform signal conditioning, analysis, and visualization. Automation and computerization in (bio)sensing encourage interdisciplinary collaborations with chemists, engineers, clinicians, and other experts, and lead to the development of novel autonomous health monitoring and diagnostics systems, among others. Automation of sensing offers remedies for environmental challenges because it enables monitoring the origins of pollutants, thereby facilitating evidence-based decision-making. Overall, we think that the sensing systems developed in the coming years will incorporate increasing number of automated and computerized features to address issues such as sampling, sample delivery, sample processing, fluid control, troubleshooting, and detection. This progress will be supported by the increasing availability of tools for mechanical and electronic prototyping (e*.*g., robotics, open-source electronics) as well as AI-based software.
